# Estimation of the Probability of Reinfection With COVID-19 by the Susceptible-Exposed-Infectious-Removed-Undetectable-Susceptible Model

**DOI:** 10.2196/19097

**Published:** 2020-05-13

**Authors:** Alexander Victor Okhuese

**Affiliations:** 1 Department of Mathematics Nasarawa State University Keffi Keffi Nigeria

**Keywords:** infectious, disease, reinfection, model, math, COVID-19, coronavirus, pandemic, outbreak, SEIRUS

## Abstract

**Background:**

With the sensitivity of the polymerase chain reaction test used to detect the presence of the virus in the human host, the worldwide health community has been able to record a large number of the recovered population.

**Objective:**

The aim of this study was to evaluate the probability of reinfection in the recovered class and the model equations, which exhibits the disease-free equilibrium state for the coronavirus disease.

**Methods:**

The model differential equation was evaluated for the disease-free equilibrium for the case of reinfection as well as the existence and stability criteria for the disease, using the model proportions. This evaluation shows that the criteria for a local or worldwide asymptotic stability with a basic reproductive number (*R*_0_=0) were satisfied. Hence, there is a chance of no secondary reinfections from the recovered population, as the rate of incidence of the recovered population vanishes (ie, *B*=0).

**Results:**

With a total of about 900,000 infected cases worldwide, numerical simulations for this study were carried out to complement the analytical results and investigate the effect that the implementation of quarantine and observation procedures has on the projection of further virus spread.

**Conclusions:**

As shown by the results, the proportion of the infected population, in the absence of a curative vaccination, will continue to grow worldwide; meanwhile, the recovery rate will continue slowly, which means that the ratio of infection rate to recovery rate will determine the death rate that is recorded. Most significant for this study is the rate of reinfection by the recovered population, which will decline to zero over time as the virus is cleared clinically from the system of the recovered class.

## Introduction

The coronavirus disease (COVID-19) pandemic has had a major impact on the global economy and on behavioral practices of people worldwide. Until its early detection in Wuhan, China in 2019, the virus was unknown to the scientific world, and the extent of its damage was unmeasurable. However, upon its outbreak, various research, including but not limited to Victor [1] and Batista [2], began to predict the scale that the virus would hit the world; the ratio of the death to recovery rate has seemingly been a positive proportion. With the slow but deliberate efforts by governments of developed and developing countries to control, slow, and possibly halt the further spread of the virus, contact tracing and testing has reached millions of people. With the sensitivity of the testing approach, the polymerase chain reaction (PCR), the infected and exposed populations were easily identified for isolation and quarantine, respectively, in a bid to slow the curve of secondary infections and manage the critically affected infected group. Meanwhile, a common trend that seems to show a ray of hope in the fight against the coronavirus was the unattended recovery of infected and exposed patients, and, despite the absence of a Food and Drug Administration-approved vaccine, this recovery rate seems to be encouraging. However, as the recovery rate and infection rate continues to increase, the question that has eluded health care workers, the Centers for Disease Control and Prevention, and the World Health Organization (WHO) is if there will be reinfection after a patient with COVID-19 has recovered clinically?

In the literature (Victor [1], Nesteruk [3], and Ming et al [4]), focus has been placed on the outbreak, exposure, and the rate of infection for COVID-19 by the use of various models to study the trend of the pandemic. In their studies, Nesteruk [3] and Ming et al [4] used the popular susceptible-infectious-removed (SIR) model to obtain optimal values for the model parameters for use with a statistical approach and, hence, predicted the number of infected, susceptible, and removed persons over time. This model approach by Nesteruk [3] has been a major breakthrough in modelling disease control and has been used by several authors (eg, Ming et al [4] and Victor [1]). However, although there exists a worldwide interest in contact tracing, testing, isolating those that are exposed to COVID-19, and estimating and projecting the rate of worldwide infections, what is more interesting is an estimation that could evaluate the probability of reinfection by those who have recovered from COVID-19. Therefore, in this study, the approach developed by Victor [1] based on an age-structured model developed and used by Victor and Oduwole [5] for HIV/AIDS transmission in Africa was adopted, which is a deterministic endemic susceptible-exposed-infectious-removed-undetectable-susceptible (SEIRUS) model.

The SEIRUS model was used due to the resulting solutions that captured the relevant parameters for the exposed and untransmitable classes, which are not present in the SIR model as used by Nesteruk [3] and Batista [2].

The resulting equations from the SEIRUS model are a system of coupled homogenous differential equations used to capture the susceptible rate, rate of exposure, infectious rate, and the rate of recovery. In addition, the equations capture the rate of reinfection, which is captured in the undetectable class that is clinically ascertained by the PCR testing approach for the recovered population.

Numerical experiments, with relevant simulation showing how the variation of the reproductive number (R_0_) affects the number of infected individuals, were carried out as well as a projection for the rate of reinfection by the recovered class. Conscious effort to evaluate the new deterministic SEIRUS model was done to reduce the R_0_ to zero and possibly halt the spread of the disease, thereby leading to an endemic equilibrium and eradication of the disease in the future.

The worldwide COVID-19 pandemic and the lack or inefficiency of purposeful and result-based interventions are great calls for other empirical and scientific interventions that seek to review strategic models and recommendations of social and scientific research for disease control. Although previous studies have been tailored toward the epidemiology and the disease-free equilibrium (DFE) where the R_0_ of the infectious population is at its bare minimum, this study seeks to evaluate the impact of a new endemic deterministic model on the endemic equilibrium while taking into consideration the possibility of the recovered population being undetectable and fit to be moved to the susceptible class, which will, therefore, imply zero secondary infection of the disease worldwide.

In summary, this study aims to use the new deterministic endemic SEIRUS compartmental model for COVID-19 dynamics, which combines quarantine and observation procedures, and behavioral change and social distancing in the control and eradication of the disease in the most exposed subpopulations to predict the chances of reinfection by the recovered class.

## Methods

### Model Variables and Parameters

As suggested in Victor [1] and Victor and Oduwole [5], the variables and parameters for the investigation of the stability analysis of the equilibrium state for the new deterministic endemic model are given in Tables 1 and 2.

**Table 1:** The variables for the new deterministic endemic model.

Variable	Description
*S*(*t*)	Number of susceptible population at time *t*
*E*(*t*)	Number of exposed population at time *t*
*I*(*t*)	Number of infected population at time *t*
*R*(*t*)	Number of infected population quarantined and expecting recovery at time *t*
*U*(*t*)	Number of recovered adults satisfying undetectable criteria at time *t*

**Table 2:** The parameters for the new deterministic endemic model.

Parameter	Description
*μ*	Natural death rate of the population
*α*_0_	Maximum death rate due to coronavirus disease (α≤α_0_)
α	Death rate of the infected population due to coronavirus disease
*φ*	Disease induced death rate of infected population not quarantined
*ϖ*	Disease induced death rate of infected receiving quarantine
*T*	Maximum lifespan after infection (*T*≥14 *days*)
*k*	Efficacy of quarantine (0≤*k*≤1)
*ρ*	Rate of recovery
*β*	Rate of transmission
*σ*	Proportion of infected population in quarantine per unit time (treatment rate)
*π*	Proportion of population from susceptible to exposed/latent class
*ε*	Proportion of removed population still being observed and being moved to susceptible class
*B*(*t*)	Incidence rate or force of infection in the population

### Model Assumptions

The following assumptions, as suggested in Victor [1] and Victor and Oduwole [5], help in the derivation of the model:

1. There is no emigration from the total population and there is no immigration into the population. A negligible proportion of individuals move in and out of the population at a given time.
2. Maturation (or maturity) is interpreted as the period between infection and symptom observation (days 1-14).
3. The susceptible population are first exposed to a latent class where they can be infected or not.
4. Some infected individuals move to the removed class when they are quarantined for observation procedures.
5. The recruitment from the S class into the E class is through contact with populations in the I class to the S class.
6. The recruitment into the R class from the I class is at a rate of σ.
7. The recruitment into the U class from the R class depends on the effectiveness of the quarantine and observation procedures at a rate of ρ.
8. Death is implicit in the model, and it occurs in all classes at a constant rate μ. However, there is an additional death rate in the I and R classes due to infection for both juvenile and adult subpopulations, denoted by φ and ϖ, respectively.

### Model Description

This study uses the deterministic endemic model where a susceptible class is a class that is yet to be infected but is open to infection as interactions with members of the *I* class continue. An infected individual is one who has contracted the coronavirus and is at some stage of infection. A removed individual is one that is confirmed to have the virus with its expected symptoms and is under quarantine while following relevant observation procedures. A member of the undetectable class is one that has been removed, does not secrete the virus anymore, and has satisfied the WHO standard to be in the undetectable class.

The following diagram [1] describes the dynamic of the SEIRUS framework and will be useful in the formulation of model equations:


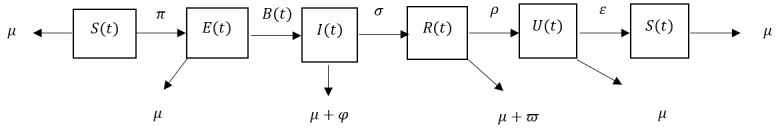


### The Model Equations

The following equations are a system of coupled homogenous differential equations for projecting the detection rate of the presence of the virus in the clinically prescribed recovered population based on the assumptions and the flow diagram previously mentioned:


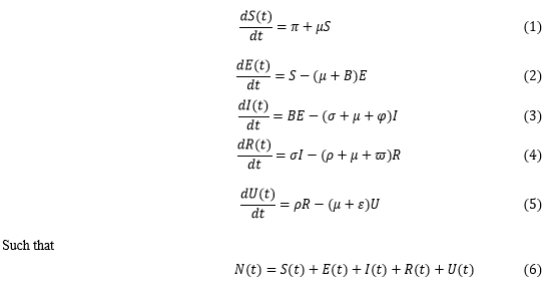


The incidence rate or force of infection at time *t*, denoted by *B*(*t*), in the population

is given by:


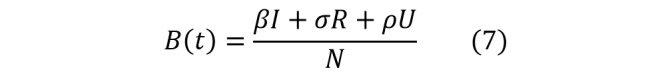


### Model Equations in Proportions

The model equations in proportion according to Victor [1] was adopted for this study as follows:


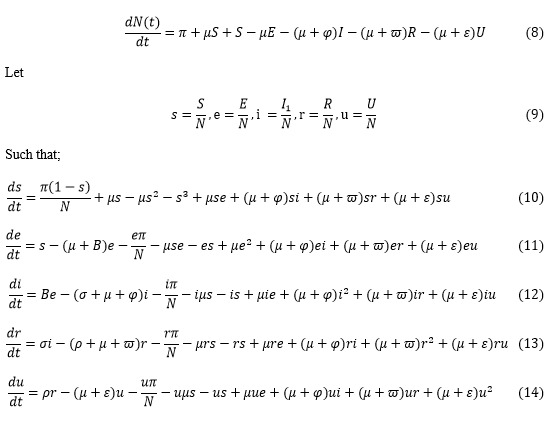


However, *s* + *e* + *i* + *r* + *u* = 1

Equations 10-14 are the model equations in proportions, which define the prevalence of infection.

### Existence and Uniqueness of a Disease-Free Equilibrium State in the SEIRUS Model

The DFE state of the endemic SEIRUS model is obtained by setting the left-hand sides of equations 10-14 to zero while setting the disease components *e* = *i* = *r* = *u* = 0, leading to equations 15 and 16.


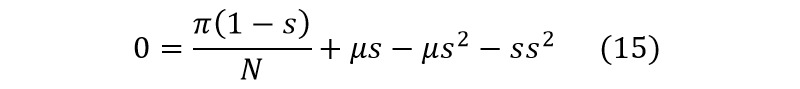


0=*s* **(16)**

After substituting equation 16 into 15 we have: 
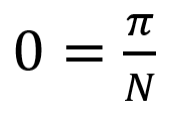
, which makes 0=*π*.

We then take 15, where *s*=0 or:

0 = *μ* – *μs* – *s*^2^ **(17)**

Simplifying this further gives us:

*As*^2^ + *Bs* + *Cμ* = 0 **(18)**

In equation 18, *A*=1, *B*=*μ*, and *C*=–*μ*.


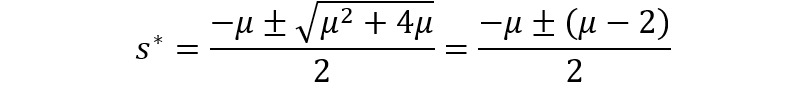


Therefore, the solution for the equations in 18 are given by:


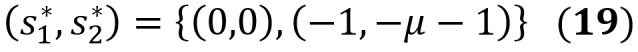


Ignoring the native values of 
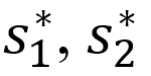
, and other stringent conditions, there exists a unique, trivial, and DFE state at (
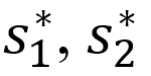
) given by (0,0). The solution of equation 19 satisfies equation 18 identically.

### Stability Analysis of Disease-Free Equilibrium State for the Recovered Population

In the event that patients recover from COVID-19, it is assumed that they are disease free for at least 14 days after their last clinical test shows that they have clinically recovered from the virus. Hence, to study the behavior of the equations 10-14 around the DFE state, *E*_0_=(0,0,0,0,0), we resort to the linearized stability approach from Victor [1], which gives us a Jacobian 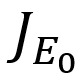 transformation of the form:


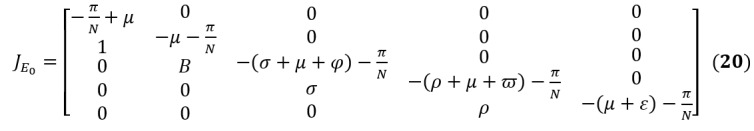


Hence, according to Gerald [6], the determinant of the Jacobian matrix 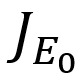 is given by the recursive definition of a 5 x 5 matrix defined as:


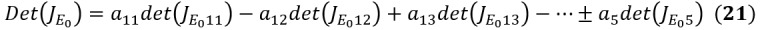


From equation 20:

*Det*(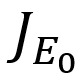)>0 **(22)**

Similarly from the Trace of the Jacobian matrix 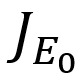 given in equation 20, we have:


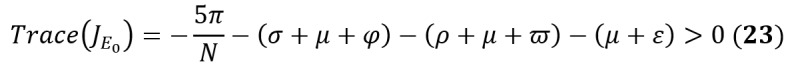


Hence, since *Def*(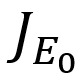)>0 and *Trace*(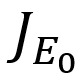)<0, which does satisfy the prescribed threshold criteria based on Gerald [6], then the DFE (*E*_0_) for COVID-19 does satisfy the criteria for a local or worldwide asymptotic stability for the recovered population. This implies that the pandemic of COVID-19, as declared by WHO [7], does not have a curative vaccine so far, and precautionary measures are advised through quarantine and observation procedures. Therefore, for the recovered population, the chances of reinfection appear to be uncertain though nearly impossible, unless regular clinical tests are not accurately administered.

### Computation of the Basic Reproductive Number of the Model

The basic *R*_0_ is defined as the number of secondary infections that one infectious individual would create over the duration of the infectious period, provided that everyone else is susceptible. *R*_0_=1 is a threshold, and if the number is below it, the generation of secondary cases is insuﬃcient to maintain the infection in human communities. If *R*_0_<1, the number of infected individuals will decrease from one generation to the next, and the disease dies out; if *R*_0_>1 the number of infected individuals will increase from one generation to the next, and the disease will persist.

To compute the basic reproductive number (*R*_0_) of the model with the incidence rate for the recovered population assumed to vanish, such that *B*=0, we employed the next generation method as applied by Deikmann et al [8] and van den Driessche and Watmough [9].


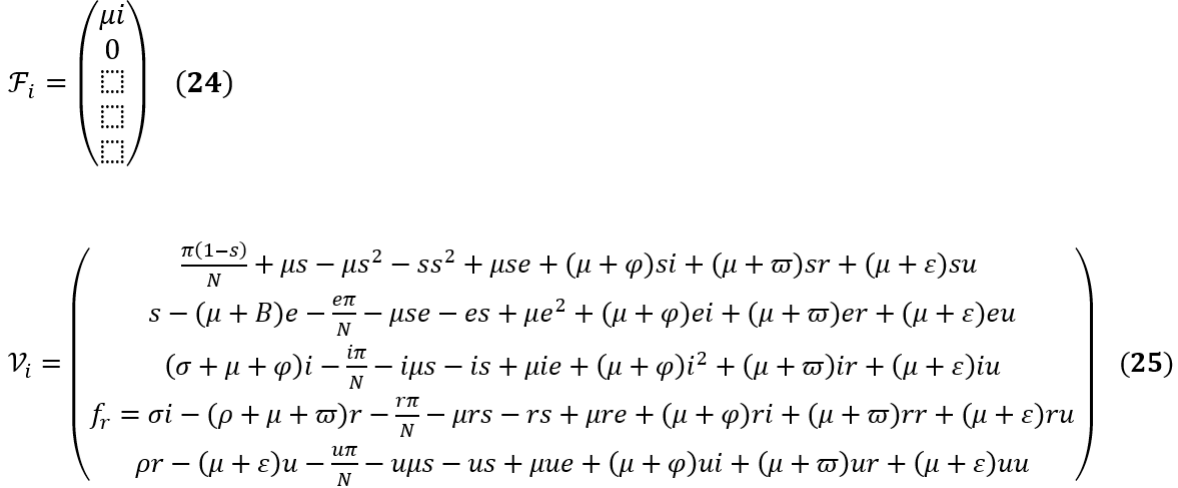


*F_i_* and *V_i_* are the rate of appearances of new infections in compartment *i* and the transfer of individuals into and out of compartment *i* by all means, respectively. Using the linearization method, the associated matrices at DFE (*E*_0_) and after taking partial derivatives as defined by:


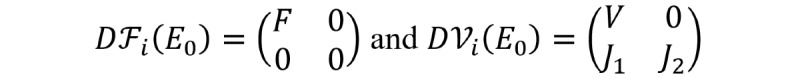


*F* is nonnegative, and *V* is a nonsingular matrix in which both are the *m* x *m* matrices defined by:


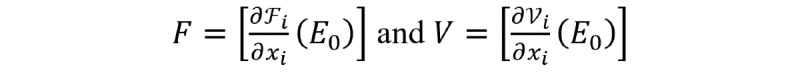


Here, 1≤*i*, *j*≤*m*, and *m* is the number of infected classes. In particular, *m*=2, and we have:


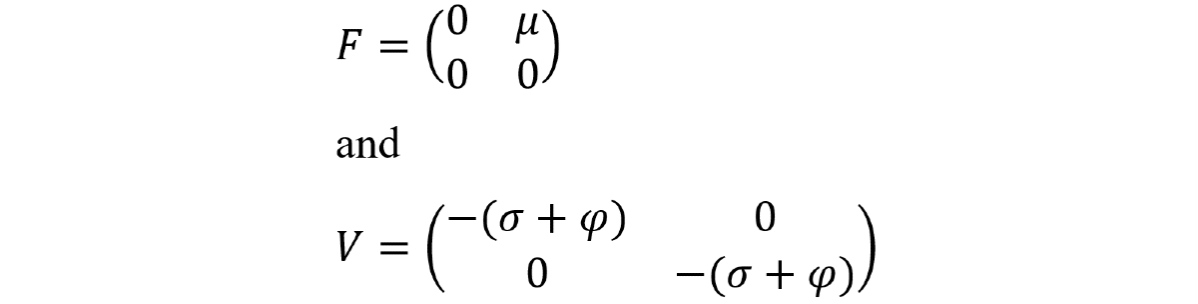


The inverse of *V* is given as:


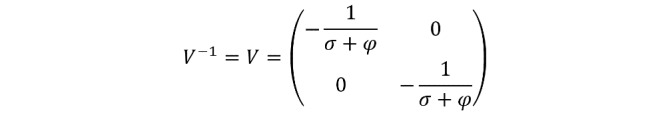


The next matrix will then be denoted by *FV*^–1^, given as:


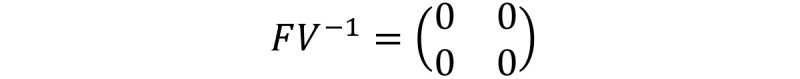


We find the eigenvalues of *FV*^–1^ by setting the determinant |*FV*^–1^ – *γI*| = 0


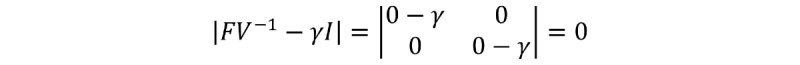


The characteristics polynomial is:

*ρ*(*γ*)=*γ*^2^

The characteristics equation is given as:

*γ*^2^=0

We solve the characteristics equation for the eigenvalues *γ*_1,2_, where *R*_0_ is the maximum of the two eigenvalues *γ*_1,2_. Hence, the basic R_0_ is the dominant eigenvalues of *FV*^–1^. Thus, we have that:

*R*_0_=0 **(26)**

The basic reproductive number (*R*_0_=0) of equation 26 shows that, with no incidence rate in the recovered population, there is no chance of a secondary infection by patients with COVID-19 who have been clinically declared negative and free from the virus (ie, the virus is completely cleared from their system). Hence, although there currently exists no clinical vaccine for the cure of COVID-19, with equation 26, there is a high chance of zero cases of reinfection after clinical recovery from the virus. 

## Results

### Description and Validation of Baseline Parameters for Worldwide Cases of COVID-19

**Figure 1:**
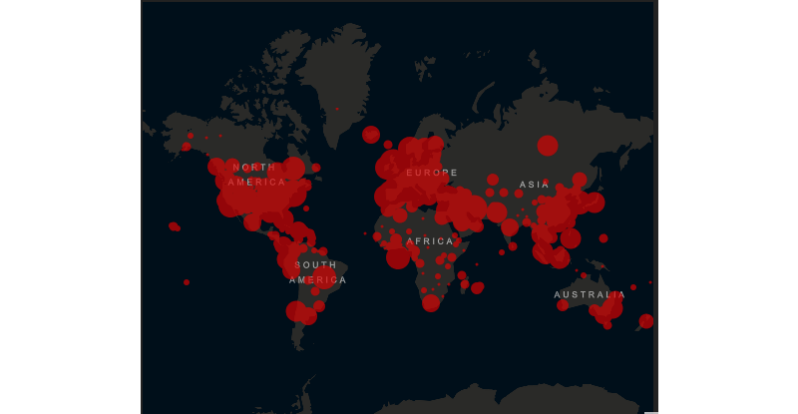
A world map showing the number of cases for each country with a coronavirus disease case.

**Figure 2:**
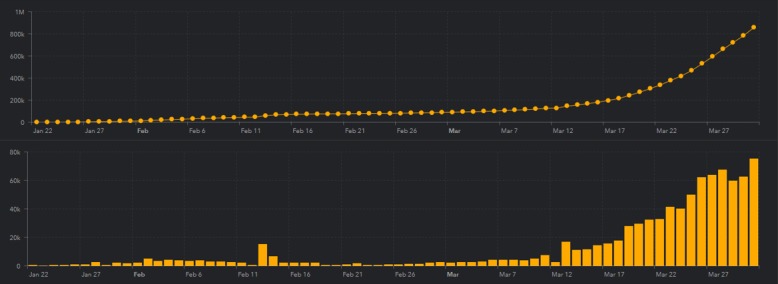
A cumulative case chart showing the number of cases of coronavirus disease.

According to the WHO [10], the total cases of COVID-19 worldwide stands at about 900,000, with a total of about 190,000 recovered, and the current total deaths is about 44,000 from about 172 countries. Figures 1 and 2 show the cumulative case count per country [11] and worldwide [11], respectively.

### Numerical Experiments of the Model

The age-structured deterministic model in equations 10-14 was solved numerically using the Runge-Kutta-Fehllberg fourth to fifth order method and implemented using Maple Software (Maplesoft). The model equations were first transformed into proportions, thus, reducing the model equations to 10 differential equations. The parameters used in the implementation of the model are shown in Table 3. Parameters were chosen in consonance with the threshold values obtained in the stability analysis of the DFE state of the model.

**Figure 3:**
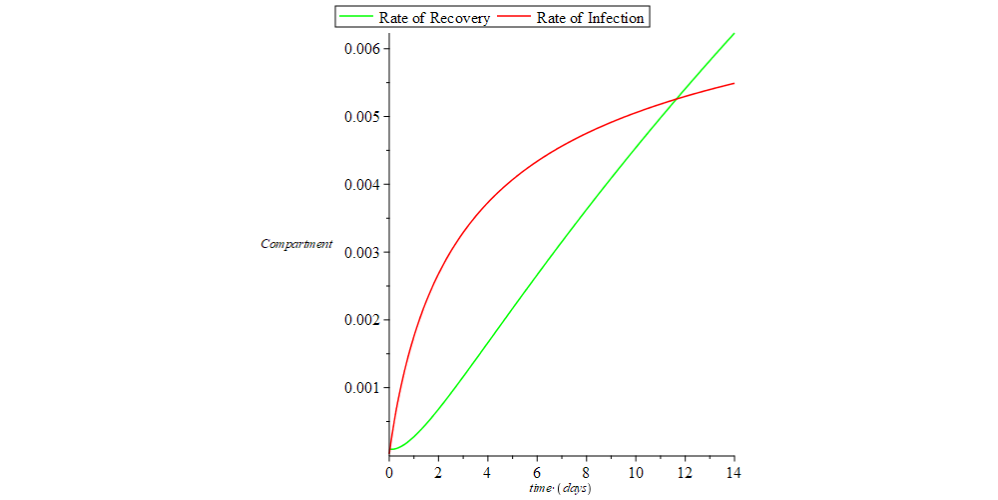
Chart of recovered and infectious compartments for coronavirus disease.

**Figure 4:**
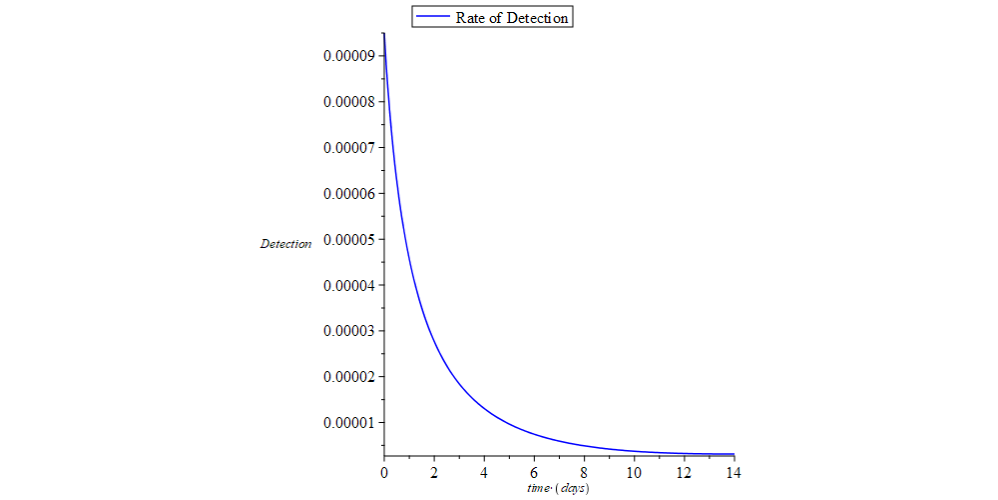
Chart of the rate of reinfection of the recovered compartment from coronavirus disease.

Hence from equation 26, the reproductive number *R*_0_=0 means there is a 100% chance of zero secondary reinfections from the recovered compartment of the COVID-19 patient group when a reinfected population interacts by contact with the susceptible population. Figure 3 shows the rate of recovery and rate of infection for COVID-19, and Figure 4 shows the rate of reinfection.

**Table 3:** Estimated values of the parameters used in the numerical experiments.

Parameters	Values	Data source	Parameters	Values	Data source
*N* (0)	7.57 billion	WPR^a^ [12]	*φ*	0.000005^b^	Assumed
*N*(1)	845,292	WHO^c^ [10]	*ϖ*	0.0000007	JHU^d^ [11]
*s*(0)	1.0000	Estimation	*T*	14 days	WHO [10]
*e*(0)	1.0000	Estimation	*k*	0.5^b^	Assumed
*i* (0)	0.00002	WHO [10]	*ρ*	0.000095	JHU [11]
*r*(0)	0.000095	JHU [11]	*β*	0.00002	WHO [10]
*u*(0)	0.000095	JHU [11]	*σ*	0.28404^e^	Estimated
μ	0.000001	WPR [12]	*π*	0.00567^b^	Assumed
*α*_0_	0.000011	Nesteruk [3]	*ε*	0.000095	JHU [11]
N/A^f^	N/A	N/A	*B*(*t*)	0.00000	Assumed

^a^WPR: World Population Review.

^b^Assumed: Hypothetical data used for research purposes.

^c^WHO: World Health Organization.

^d^JHU: Johns Hopkins University.

^e^Assumed: Based on Victor [1], Batista [2], and Nesteruk [3].

^f^Not applicable.

## Discussion

### Principal Findings

The analysis clearly shows that the secondary infection rate satisfies the local and worldwide stability criteria and the DFE for an endemic situation. Unlike the respiratory syncytial virus, which causes a significant respiratory disease often in those 5 years or younger, COVID-19 is estimated to burden more than 10,000 people worldwide. Although the stability analysis shows that there is no chances of secondary reinfection by the recovered class, the rate of the infectious will continue to rise asymptotically over a long period of time and there after begin to slide in a normal trajectory if no vaccine is available. Batista [2] and Nesteruk [3] focused their study on the impact of the infectious class in the subpopulation with the SIR model and forecasted a rapid geometric growth in the spread of the virus worldwide and a subsequent progression in the rate of recovery among the exposed and infectious groups.

According to Victor [1], the model equations that exhibit the DFE (*E*_0_) state for COVID-19 satisfies the criteria for a local or worldwide asymptotic stability when the basic *R*_0_=0 for an endemic situation. This implies that the COVID-19 pandemic, as declared by WHO [7], does not have a curative vaccine yet, and precautionary measures are advised through quarantine and observation procedures.

However, with the various make shift treatments, social distancing measures, and quarantine strategies being adopted, the recovery rate will keep rising slowly but steadily over a long period of time. Therefore, as the recovery rate continues to grow steadily, the number of recovered patients who have been clinically declared free of the virus by the PCR test are also declared uninfectious as long as the virus is completely cleared from their system, and the rate of detection will vanish, making the rate of secondary infection *R*_0_=0 as long as the incidence rate *B*=0.

### Conclusions

There is a need for a dedicated effort from individual populations, governments, health organizations, policy makers, and stakeholders. The world is hardly rid of COVID-19, and further spread is eminent; the rate of infection will continue to increase despite the increased rate of recovery until a curative vaccine is developed.

With the worldwide health sector in a bid to tackle COVID-19, this study gives encouragement to the policy makers and public health care sectors, as there is zero secondary reinfections by the recovered population. Therefore, the policy makers and public health sectors can enhance contact tracking, tracing, and testing to improve the isolation and quarantine of the infected and exposed classes. In addition, the health sector could use COVID-19 antibodies from the samples of the recovered class to develop effective vaccines for the virus. However, since the hypothesis of zero reinfections has not been clinically proven, further observations should be carried out on the recovered class in clusters to study the progression of the exposed with the re-exposed subpopulations to see, by clinical examination, the possibilities of reinfection and, thereby, promote the use of these antibodies for vaccine creation.

### Limitation

This study was limited by the variability of data available at the time of developing this paper. Meanwhile, from the statistics, the infected cases and fatalities were projected to increase geometrically. Therefore, the findings of this study are based on sample data taken at the time of the study.

In addition, with the SEIRUS model and the discovery that the *R*_0_=0, we concluded that there are no secondary reinfections from the recovered population, as the rate of incidence of the recovered population vanishes. However, reports from worldwide public health data have shown that there has been a few rare cases of reinfection of some from the recovered class, and they are suspected to be reinfected by a rare type of the coronavirus but not COVID-19.

## Abbreviations

COVID-19: coronavirus disease
DFE: disease-free equilibrium
PCR: polymerase chain reaction
R0: reproductive number
SEIRUS: susceptible-exposed-infectious-removed-undetectable-susceptible
SIR: susceptible-infectious-removed
WHO: World Health Organization

## References

[1] Victor A (2020). Mathematical predictions for COVID-19 as a global pandemic. SSRN Journal.

[2] Batista M (2020). Estimation of the final size of the coronavirus epidemic by the SIR model. ResearchGate.

[3] Nesteruk I (2020). Statistics based predictions of coronavirus 2019-nCoV spreading in mainland China. medRxiv.

[4] Ming W-K, Zhang CJP (2020). Breaking down of healthcare system: mathematical modelling for controlling the novel coronavirus (2019-nCoV) outbreak in Wuhan, China. bioRxiv.

[5] Victor AO, Oduwole HK (2020). Evaluating the deterministic SERIUS model for disease control in an age-structured population. Global Sci J.

[6] Gerald T (2012). Ordinary Differential Equations and Dynamical Systems.

[7] (2020). World Health Organization.

[8] Diekmann O, Heesterbeek JAP, Roberts MG (2010). The construction of next-generation matrices for compartmental epidemic models. J R Soc Interface.

[9] van den Driessche P, Watmough J (2002). Reproduction numbers and sub-threshold endemic equilibria for compartmental models of disease transmission. Math Biosci.

[10] (2020). World Health Organization.

[11] (2020). Johns Hopkins University.

[12] World Population Review.

